# Leucine-rich glioma-inactivated protein 1 antibody-associated encephalitis in a 22-month-old girl: a case report

**DOI:** 10.1186/s12887-023-04191-y

**Published:** 2023-08-08

**Authors:** JiaChang Zhao, XiaoMing Yu, GuangLi Qu, ShuQi Wang, YanJun Wang

**Affiliations:** Department of Pediatrics, Affiliated Hospital of ChiFeng University, ChiFeng, Inner Mongolia Autonomous Region China

**Keywords:** Autoimmune encephalitis, LGI1, Children, Seizures

## Abstract

**Background:**

LGI-1 antibody-associated encephalitis is a type of autoimmune encephalitis with a lower prevalence than NMDAR antibody-associated encephalitis. LGI-1 antibody-associated encephalitis is the second most prevalent of all autoimmune encephalitides. LGI-1 antibodies interfere with the interactions of inter-synaptic proteins to produce clinical manifestations (N Engl J Med 378:840–851, 2018).

**Case presentation:**

Leucine-rich glioma-inactivated protein 1 (LGI-1) antibody-associated encephalitis is a subtype of autoimmune encephalitis with a low incidence. We report a case of a girl aged 22 months with convulsive seizures, psycho-behavioral abnormalities, sleep disorders, and limb tremors. This patient was diagnosed with LGI-1 antibody-associated encephalitis based on electroencephalography (EEG) examinations and autoimmune encephalitis antibody analyses. A combined therapy of anti-epileptic and immunosuppressant drugs was effective in controlling the patient’s neurological symptoms.

**Conclusions:**

The incidence of LGI-1 antibody-associated encephalitis is low and it occurs mostly in middle-aged and elderly patients, although it occasionally occurs in pediatric patients. To the best of our knowledge, this report describes the youngest patient with LGI-1 antibody-associated encephalitis. Following timely diagnosis, administration of anti-epileptic and immunosuppressant therapy was remarkably effective.

## Background

Autoimmune encephalitis (AE) is an encephalitis mediated by an autoimmune response. *N*-methyl-d-aspartate receptor (NMDAR) antibody-associated encephalitis was first identified in 2007, followed by leucine-rich glioma inactivated protein 1 (LGI-1) antibody-associated encephalitis and anti γ-aminobutyric acid type B receptor (GABABR) antibody-associated encephalitis [1, 2]. The characteristic symptoms of autoimmune encephalitis are seizures, memory loss, and neuropsychiatric disturbances [3].

In this case report, we describe a 22-month-old girl who presented with convulsive seizures, psycho-behavioral abnormalities, sleep disorders, and limb tremors. This patient was diagnosed with LGI-1 antibody-associated encephalitis through a combination of electroencephalography examinations and autoimmune encephalitis antibody analyses. Ethics committee of affiliated hospital of ChiFeng University approved this study.

## Case presentation

The patient was a 22-month old girl. The family described the patient as often becoming “dazed” with no apparent reason, which manifested as a sudden stopping of movement and looking straight ahead with both eyes open for approximately 2–3 s. These episodes had been occurring approximately 4–5 times a day for the previous 2 weeks. The patient was admitted to our hospital with a diagnosis of tonicity of the limbs with loss of consciousness for approximately 5 s, occurring 5–6 times a day.

**Fig. 1:**
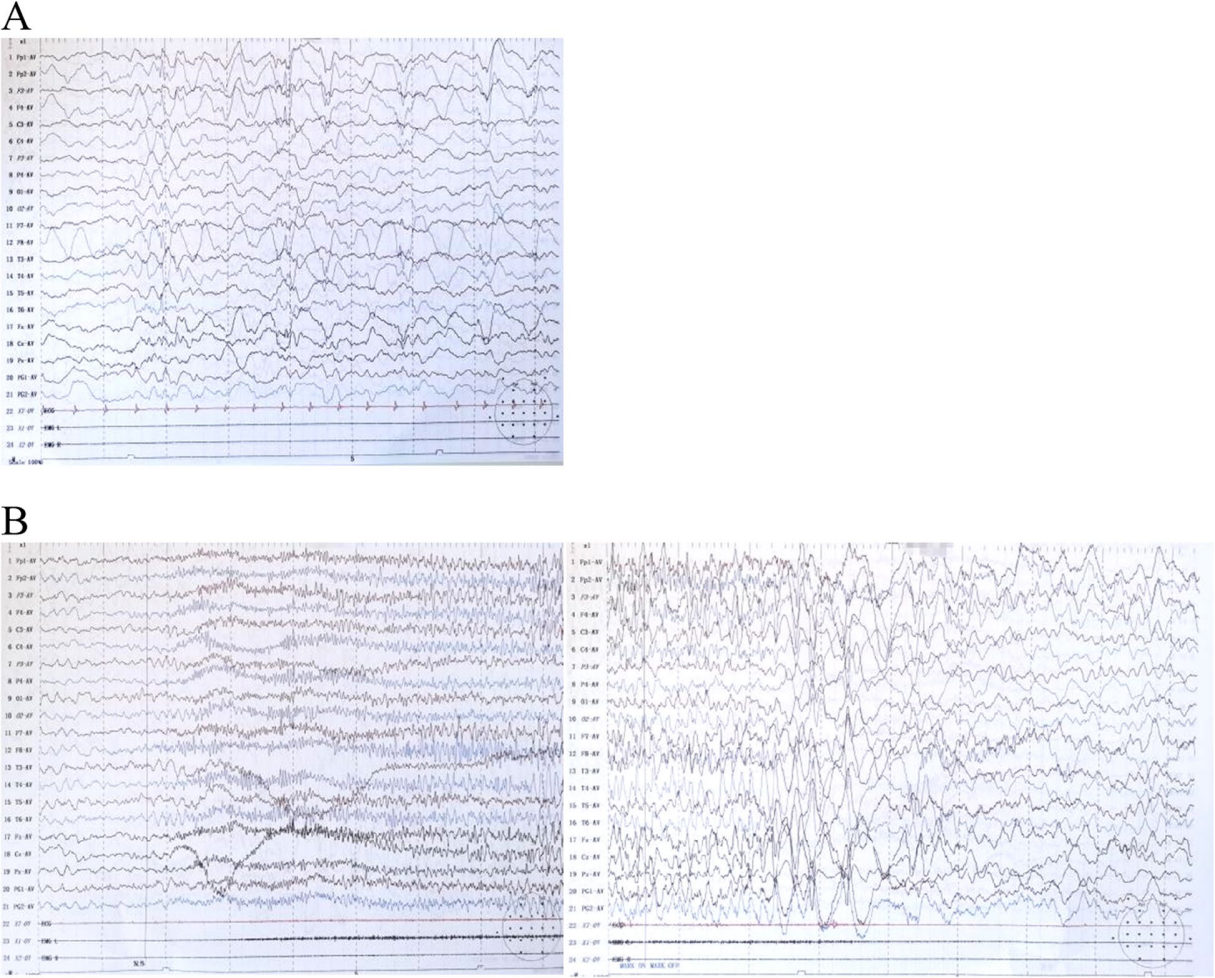
Video electroencephalography (EEG) recordings. **A** EEG background shows 5–6 Hz slow waves and activities.. Interictal EEG showed right forehead spike and sharp waves complex. **B** Ictal EEGs show low-medial amplitude β style fast rhythm gradually change to spike wave complex. It appears frequently at sleep stage with Asymmetric muscle rigidity and tremor for 10–15 s

Electroencephalography (EEG) monitoring revealed frequent convulsive seizures with loss of consciousness and tonicity of the extremities. There were a total of 33 seizures in 24 h, with significant interictal discharges (Fig. 1). No abnormalities were found in the cranial magnetic resonance imaging (MRI) scans or hippocampal scans or in blood and urine organic acid screens. The cerebrospinal fluid (CSF) pressure was normal, and the CSF cell numbers and glucose concentrations were within normal ranges. Further tests for autoimmune encephalitis antibodies were recommended, but the family declined these. The patient was treated with an anti-epileptic drug (sodium valproate). This treatment was successful, and the patient was discharged 9 days later due to the decrease of seizures.

Two weeks later, the patient was brought to our hospital again due to an increasing number of convulsive episodes. The patient exhibited tonic seizures followed by generalized flexion, which resolved after a few seconds. Repeat EEG examination findings were similar to those obtained at the first admission. The patient was readmitted to the hospital.

The patient had begun to exhibit memory loss 3 months before the second admission, which was first characterized by a reduced ability to understand what was represented by images in picture books. The patient subsequently began to exhibit irritability that later developed into frequent episodes of hitting and biting. Family history-taking revealed that 3 months before the patient’s second admission, the patient’s grandfather had begun to exhibit personality changes, mainly in the form of irritability, accompanied by limb tremors and memory loss. He was thus examined at Xuanwu Hospital in Beijing during the patient's second admission to our department. The patient’s personality had also gradually changed, accompanied by nocturnal sleeplessness, and the patient was not able to communicate with her family at the time of her second admission. Blood tests did not detect a certain viral infection. We recommended patients undergoing a genomic sequencing analysis to check for virus infection. The family refused the examination for financial issue. The patient was also having more frequent and longer convulsive seizures, was exhibiting upper-limb tremors between seizure episodes, and could not sit, stand, or walk. The patient was treated with topiramate and clonazepam in combination with sodium valproate, but this failed to alleviate the patient’s neurological symptoms.

**Fig. 2:**
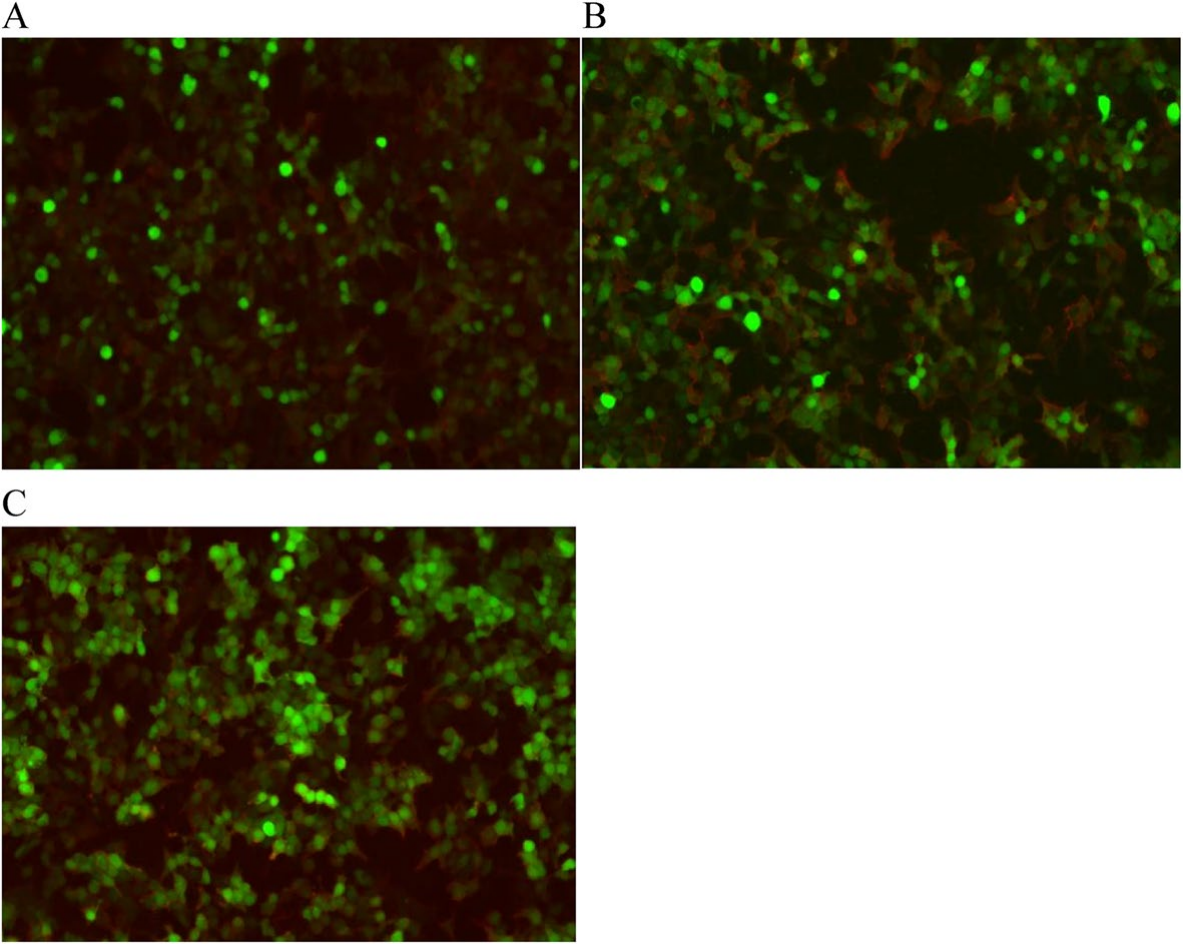
Image of positive immunofluorescence results. **A** LGI-1 IgG antibodies in CSF (1:1 +). **B** Image of positive result for LGI-1 IgG antibodies in serum (1:1000 +). **C** Image of positive result for CASPR2 IgG antibodies in serum (1:100 +). Red fluorescence

Cranial and hippocampal MRI showed no abnormality; however, this did not exclude the possibility of autoimmune encephalitis. The parents were again advised to allow an autoimmune encephalitis antibody test to be performed, and they gave their consent. The test yielded the following results positive for LGI-1 immunoglobulin G (IgG) antibody in CSF (1:1 +) and serum (1:1000 +), and positive for contactin-associated protein-like 2 (CASPR2) IgG antibody (1:100 +) in serum (note: pre-diagnosis was performed using a two-color fluorescent cytometric bead array assay). Subsequent to these results being obtained, the patient’s grandfather’s serum autoimmune encephalitis antibody test results were also found to be positive for LGI-1 and CASPR2 antibodies in serum (Fig. 2).

The patient was diagnosed with LGI-1 antibody-associated encephalitis, with the presence of a tumor having been excluded based on other ancillary tests. Human Ig and methylprednisolone shock therapy were given. The patient’s grandfather was diagnosed with Morvan syndrome and was given the appropriate immunotherapy. The patient’s family did not agree to further tests for CSF pathology, and it was therefore not possible to subject a CSF sample to second-generation sequencing to determine whether the disease was related to a viral infection.

**Fig. 3:**
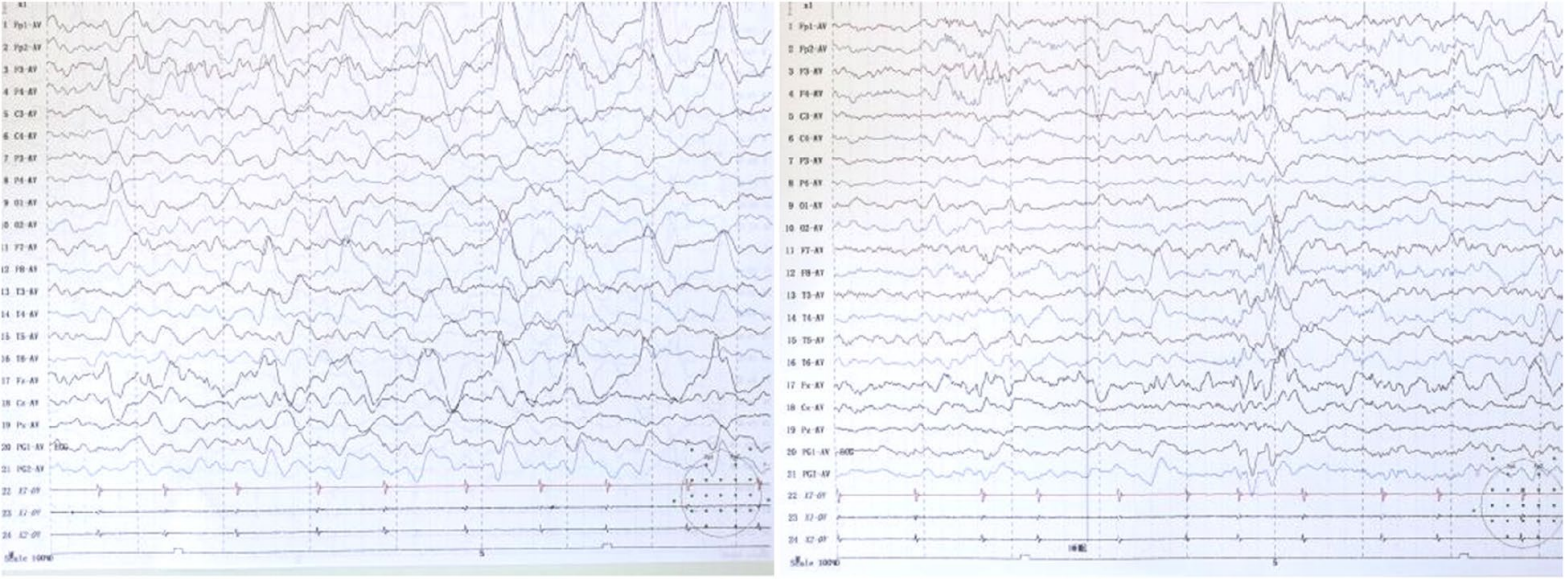
EEG recordings after 24 days, show slow wave complex and no ictal EEG was recorded

Concomitant to the delivery of methylprednisolone shock therapy, the patient’s antiepileptic drug regimen was adjusted by discontinuation of clonazepam and addition of levetiracetam. We gave methylprednisone 2 mg/kg.d orally. According to the < Chinese Expert Consensus on the Diagnosis and Treatment of Autoimmune Encephalitis > , the total course of prednisone is about 6 months, and we gradually reduce prednisone after 5.5 months. This immunotherapy and antiepileptic drug treatment led to a gradual disappearance of the patient’s convulsive seizures. After a third course of methylprednisolone shock therapy, the patient was able to communicate briefly with her family, sit without assistance, and walk with assistance without displaying convulsions. Another EEG examination was performed and the results showed that no convulsive seizures had occurred and that the interictal discharges had decreased in frequency and magnitude. (A further EEG examination was performed before discharge, Fig. 3).

**Fig. 4:**
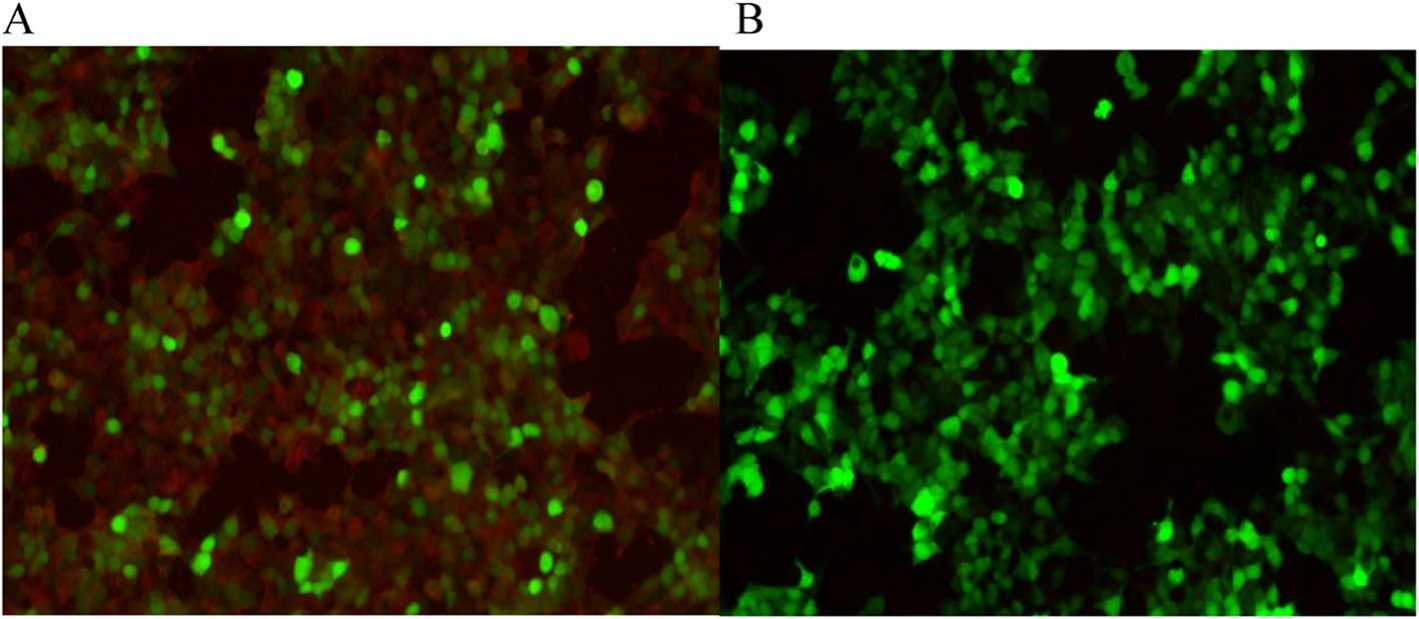
**A** Image of second positive result for LGI-1 IgG antibodies in serum (1:10 +) (red fluorescence). **B** Negative result for CASPR2 IgG antibodies in serum

Follow-up oral prednisone treatment was continued. Another serum autoimmune encephalitis antibody test was performed before discharge and was positive for LGI-1 IgG antibodies (1:10 +) and negative for CASPR2 IgG antibodies (Fig. 4) (note: pre-diagnosis, was performed using a two-color fluorescent cytometric bead array assay).

**Fig. 5:**
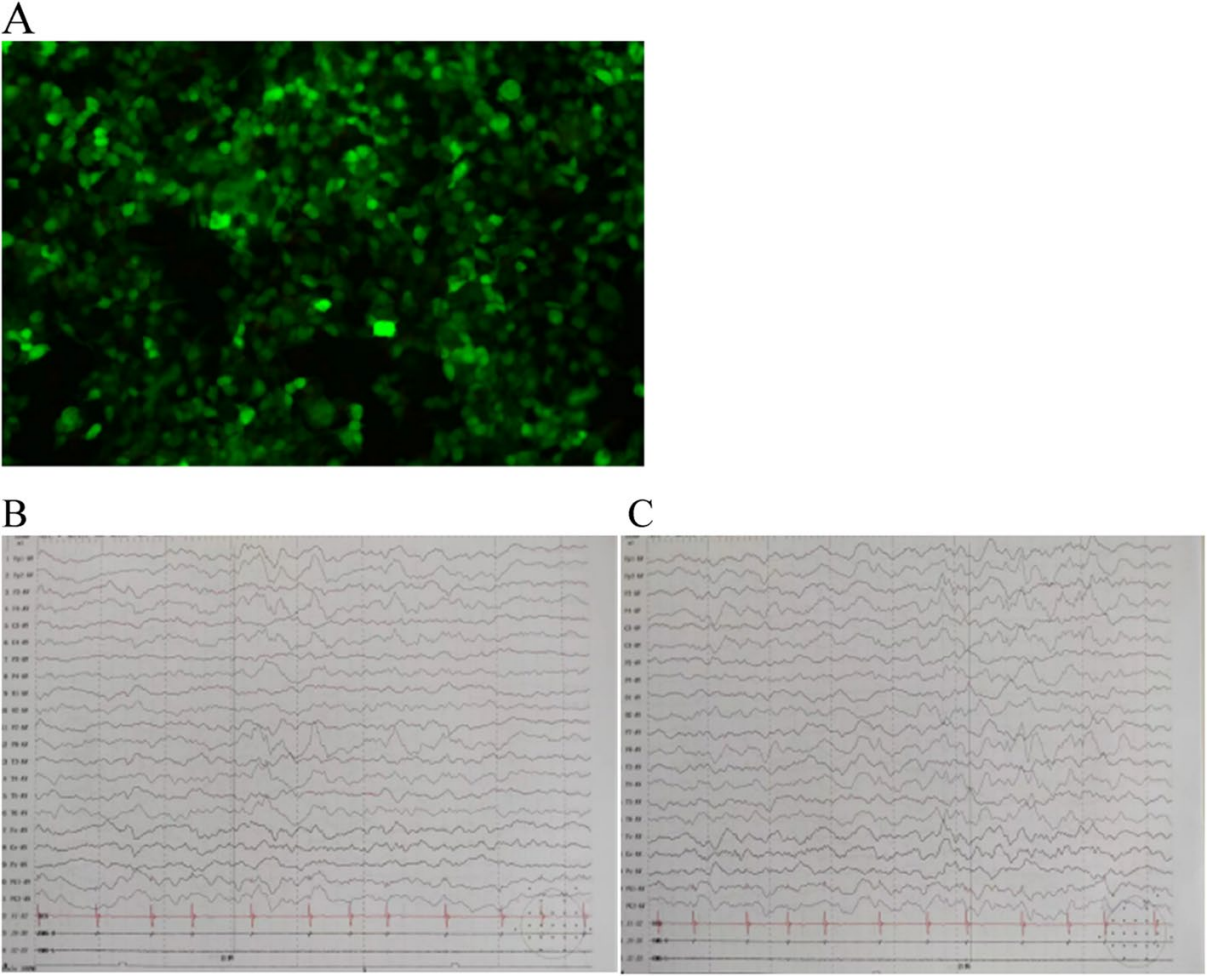
**A** Image of negative LGI1 immunofluorescence results one month after discharge. **B**,** C** No convulsive seizures were found in video EEG monitoring at one-year follow-up

The patient was discharged on the 29^th^ day of her second admission. At the time of discharge, the patient’s convulsive episodes and irritability had ceased, and her physical activity and ability to communicate with family members had returned to pre-morbid levels. One-month follow-up after discharge, the child had no seizures, no irritability, no limb tremor, and normal communication ability. Serum test showed that LGI1 antibodies (red fluorescence) were negative (Fig. 5A). No convulsive seizures were found in video EEG monitoring (Fig. 5B and C). One-year follow-up after discharge has been completed, patient did not relapse with a normal mental status and physical activity.

## Discussion and conclusions

LGI1 antibody-associated encephalitis is most commonly seen in middle-aged and elderly male patients, most of whom have an acute or subacute onset of disease. The main clinical manifestations include memory loss, abnormalities in mental behavior, and seizures. The latter is usually due to temporal lobe epilepsy, and thus epilepsy caused by this disease is poorly treated with antiepileptic monotherapy. Facio-brachial dystonic seizures (FBDS) are another characteristic manifestation of the disease and present as frequent, transient dystonia-like seizures in the unilateral arms and face or lower extremities. Some patients also have abnormal sensory aura, dystonia, speech disorders, limb tremors, sleep disorders and cerebellar ataxia, and may also have intractable hyponatremia [3].

LGI-1 antibody-associated encephalitis is rarely seen in children, in whom typical FBDS symptoms have not been observed. Pediatric patients may present with seizures, sleep disturbances, mental irritability, and limb tremors. The disease rarely occurs in combination with tumors, and may be accompanied by thymoma in approximately 5–10% of middle-aged and elderly patients [4–6]. The low incidence of the disease in children means that tumors associated with the disease have not been seen in pediatric patients. It remains unclear whether the disease is related to immune damage caused by viral infection.

The sera of the patient and her grandfather contained LGI-1 and CASPR2 antibodies, with concentrations of the former being higher than those of the latter. In addition, both patients exhibited psychiatric symptoms, personality changes, and limb tremors, demonstrating that both had the disease at the same time. As they lived in the same environment, simultaneous viral infection might have caused the disease; however, the patient’s family did not agree to further second-generation genetic sequencing, and thus this possibility could not be explored.

LGI-1 antibody-associated encephalitis differs from NMDAR antibody-associated encephalitis in the following ways. 1) NMDAR antibody-associated encephalitis accounts for approximately 80% of cases of autoimmune encephalitis, and its incidence is significantly higher than that of LGI-1 antibody-associated encephalitis. 2) NMDAR antibody-associated encephalitis is more common in children and young women, whereas LGI-1 antibody-associated encephalitis is more common in middle-aged and elderly males and is rarely seen in children. 3) Both diseases can present with epilepsy and psychiatric symptoms, but NMDAR antibody-associated encephalitis has more prodromal manifestations such as fever and headache, more commonly causes involuntary movements, and has a relatively more acute onset. 4) Different antibodies detected in CSF and serum.

Immunotherapy is effective for LGI1 antibody-associated encephalitis. The first-line treatment options are intravenous immunoglobulin, glucocorticoids, and plasma exchange. Second-line drugs such as cyclophosphamide and rituximab are available for those with poor results. For those with unsatisfactory results or recurrent disease, a long course of immunotherapy is available, with morte-macrolimus as the main drug [7].

This patient had satisfactory results with immunotherapy and combined antiepileptic drugs. At the time of discharge, the patient was seizure-free, had no limb tremors, had fully recovered limb mobility, had no irritability or sleep disturbance, and had recovered a pre-morbid level of ability to communicate with her family.

In conclusion, LGI-1 antibody-associated encephalitis is rarely seen in children. Thus, when children present with clinical manifestations such as seizures, abnormal mental behavior, memory loss, and limb tremors, further clinical investigation is required to make a diagnosis of LGI1 antibody-associated encephalitis. The disease is not effectively treated with oral antiepileptic monotherapy, and early immunotherapy is required. Unfortunately, because of the rarity of the disease in children, there is a lack of large sample studies in the literature, and only case reports are available. Follow-up case reports of this disease are critical for providing information to support large-sample or multicenter studies.

## Data Availability

All data generated or analysed during this study are included in this published article [and its supplementary information files].

## Abbreviations

LGI-1: Leucine-rich glioma-inactivated protein 1
NMDAR: N-methyl-D-aspartic acid receptor
EEG: Electroencephalography
GABABR: γ-Aminobutyric acid type B receptor
MRI: Magnetic resonance imaging
CSF: Cerebrospinal fluid
IgG: Immunoglobulin G
CASPR2: Contactin-associated protein-like 2
FBDS: Facio-brachial dystonic seizures
